# Coronary Artery Disease in CKD-G5D Patients: An Update

**DOI:** 10.31083/j.rcm2408227

**Published:** 2023-08-08

**Authors:** Pan Gao, Xingjian Zou, Xin Sun, Chun Zhang

**Affiliations:** ^1^Department of Nephrology, Union Hospital, Tongji Medical College, Huazhong University of Technology, 430022 Wuhan, Hubei, China

**Keywords:** CAD, CKD-G5D, dialysis, coronary artery disease, management

## Abstract

Patients with chronic kidney disease treated by dialysis (CKD-G5D) are characterized by a high prevalence of coronary artery disease (CAD). Such patients differ from non-uremic CAD patients and have been excluded from several clinical CAD trials. CKD-G5D patients may be asymptomatic for their CAD, making their risk stratification and management challenging. This review will focus on the incidence, epidemiology, pathophysiology, screening tools, and management/treatment of CAD in CKD-G5D patients. It will also review recent studies concerning the screening tools and management strategies available for these patients. The need for improved evaluation of cardiovascular risk factors, screening and early intervention for symptomatic CAD in CKD-G5D patients will be highlighted.

## 1. Introduction

Patients with chronic kidney disease (CKD) have a high incidence of cardiovascular disease and especially coronary artery disease (CAD), which is the leading cause of death in these patients [1, 2]. CAD is almost always the result of atherosclerosis, and coronary artery atherosclerotic plaques may rupture if they are unstable or if there is inflammation. This causes substances that promote clotting to enter the blood, resulting in the activation of platelets and leading to acute thrombosis [3, 4]. The end result is acute coronary syndrome (ACS), which includes unstable angina, non-ST-segment elevation myocardial infarction (NSTEMI), and ST-segment elevation myocardial infarction (STEMI). In addition to ACS [5, 6], the European Society of Cardiology (ESC) guidelines introduced the concept of chronic coronary syndrome (CCS) in 2019 [7]. This replaces the former concept of stable coronary heart disease, which specifically includes chronic stable exertional angina, ischemic cardiomyopathy, and a stable course after ACS. CKD is defined as a long-term, progressive decline in renal function. A glomerular filtration rate (GFR) of <15 mL/min/1.73 $m^{2}$ is referred to as CKD-G5, and patients who receive dialysis for CKD are known as CKD-G5D. These patients have more co-morbidities and develop more complications [1, 8]. This review will discuss the epidemiology, pathophysiology, diagnosis, treatment and management of CAD in CKD-G5D patients.

## 2. Epidemiology

The incidence of cardiovascular disease increases during the progression of CKD into end-stage renal disease (ESRD), with many patients dying from cardiovascular disease in the late stages [2, 8]. As the GFR declines, the probability of developing CAD increases in a linear fashion. The prevalence of CAD in hemodialysis (HD) patients aged >65 years is as high as 50% [9]. The prevalence of traditional risk factors for CAD, including diabetes, hypertension and hyperlipidemia is also significantly elevated in CKD patients [1, 2]. Patients with CKD-G5D are also exposed to other non-traditional risk factors associated with uremia, such as inflammation, oxidative stress, and abnormal calcium and phosphate metabolism [10]. Furthermore, dialysis itself plays an important role in the pathophysiology of these non-traditional risk factors [11]. Consequently, CAD is associated with a higher incidence of morbidity and mortality in patients with CKD-G5D.

## 3. Pathophysiology of CAD in CKD-G5D Patients

Cardiac damage in patients with CKD is often referred to as cardiorenal syndrome (CRS). This describes a specific acute and chronic clinical manifestation in which the heart or kidney is primarily dysfunctional, leading to a series of feedback mechanisms that result in organ damage and subsequent adverse clinical outcomes [12, 13]. The pathophysiology of CRS is complex, multifactorial, and dynamic. The CAD in CKD patients discussed in this review is also associated with CRS [14], with a large proportion of CKD-G5D patients suffering from CAD. Traditional risk factors play a leading role in the early stages of CKD, whereas non-traditional factors predominate in CKD-G5D patients [9]. It has been reported that atherosclerotic plaques in the coronary arteries of CKD patients develop faster and result in more serious events than in non-CKD patients due to a more intense inflammatory response [15]. Vascular calcification (VC) is also more common in CKD patients [16] and is associated with plaque instability and inflammation, which may lead to an ACS [9].

Non-traditional factors have a major impact on the development of CAD in patients with CKD-G5D. Chronic inflammation is a major contributor to the process of arteriosclerosis and calcification of blood vessels and can usually be detected in CKD-G5D patients. Studies have shown elevated levels of inflammatory markers in the plasma of CKD patients, while the levels of tumor necrosis factor-α (TNF-α), Interleukin-6 (IL-6) [17, 18] and other pro-inflammatory markers are also increased in CKD-G5D patients. The need for dialysis in CKD-G5D patients may also stimulate the immune system, leading to chronic inflammation [19]. Bacterial endotoxin and DNA found in the dialysate can induce production of the proinflammatory factor IL-6. Chronic inflammation in HD patients may also be caused in part by the repeated contact of blood with artificial materials in extracorporeal circuits. These have been shown to activate immunologically active cells and help maintain a chronic inflammatory state. Dialysis membranes with a large surface area may play a key role in activating this inflammatory response [20], and the dialysis catheter itself may be a source of inflammation [11]. Chronic inflammation leads to arteriosclerosis through various mechanisms. It is well known that vascular smooth muscle cells (VSMC) have a contractile phenotype in the physiological state that maintains the normal structure and function of the blood vessel wall [21]. Inflammation can induce the VSMC phenotype to transform into osteoblast-like cells, release calcified extracellular vesicles, and stimulate the progress of VC [22, 23]. Inflammation can also activate the endoplasmic reticulum stress pathway, leading to increased intake of inorganic phosphate and eventually resulting in phenotypic transformation of VSMC and mineral accumulation [24].

The kidney is one of the main sources of antioxidant enzymes, and oxidative stress is thus closely related to renal function. Oxidative stress occurs when the balance between oxidation and resistance to oxidation is reversed [25]. Excessive accumulation of reactive oxygen species (ROS) produced by cell metabolism leads to endothelial cell damage and atherosclerosis. It should be noted that dialysis also increases ROS. During HD, blood exposure to the dialysis membrane and dialysate triggers the activation of complement factors, platelets, and polymorphonuclear leukocytes, with subsequent ROS production occurring within minutes of starting the HD session [26]. Against the background of chronic inflammation present in CKD-G5D patients, increased production of the pro-inflammatory factor TNF triggers oxidative stress. This in turn leads to decreased production of endothelial nitric oxide (NO) and phenotypic transformation of VSMC, thus eventually leading to arteriosclerosis [21].

CKD-G5D patients frequently have hypercalcemia and hyperphosphatemia. High phosphate levels can directly promote VC via nuclear factor kappa-B (NF-κB) signaling [24], as well as inducing the phenotypic transformation of VSMC into osteoblast-like cells. Calcium phosphate mineral deposition inside the blood vessels of dialysis patients intensifies the development of VC and contributes to the progression of arteriosclerosis [27].

Uremic toxins in CKD-G5D patients also lead to coronary heart disease. Uric acid affects VSMC proliferation by reducing NO production. Advanced glycation end-products affect the function of endothelial nitric oxide synthase (eNOS), leading to endothelial dysfunction [28]. They can also induce the phenotypic transformation of VSMC, thereby leading to arteriosclerosis [29]. Hypercholesterolemia often promotes vascular inflammation and oxidative stress, which in turn gives rise to endothelial dysfunction and the proliferation of VSMC. Several protein-bound uremic toxins found in uremic patients, such as indoxyl sulfate and p-cresyl sulfate [14], provide another pathway for the progression of atherosclerosis in CKD-G5D patients by altering oxidative stress [30, 31].

Soluble urokinase Plasminogen Activator Receptor (suPAR) is also closely associated with the progression of atherosclerosis in patients with CKD. SuPAR is an immune-derived pathogenic factor and a common therapeutic target for kidney disease. It is also a biomarker for the occurrence of kidney disease, and its expression level is closely related to cardiovascular outcomes. Both experimental animal research and clinical data have shown that higher suPAR levels are positively correlated with more atherosclerotic plaques. Hindy *et al*. [32] found that overexpression of suPAR favored the progression of atherosclerosis. These workers hypothesized that increased suPAR expression might cause the recruitment of monocytes into the vascular wall by chemotaxis, thereby altering their function and inducing changes to the immune system. SuPAR generally acts on monocytes and myeloid cells to make them more atherosclerotic [32].

**Fig. 1.**
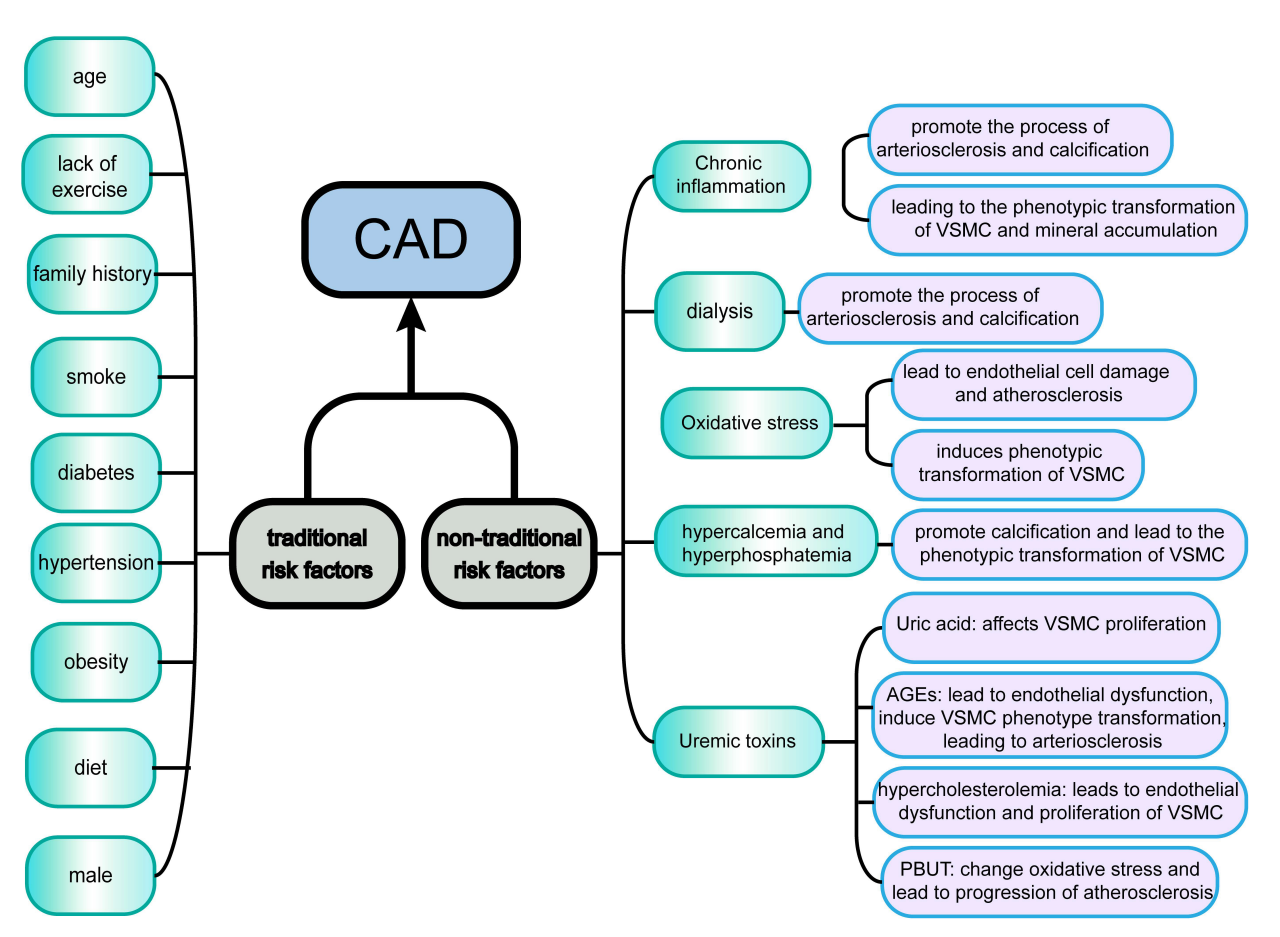
**Traditional and non-traditional risk factors for CAD in CKD-G5D patients**. Traditional and non-traditional risk factors act synergistically to cause CAD. Traditional risk factors include age, diabetes, and obesity, while non-traditional risk factors include chronic inflammation and dialysis. With the progression of CKD, there is a gradual shift from traditional risk factors to non-traditional risk factors. Abbreviations: CAD, coronary artery disease; CKD, chronic kidney disease; CKD-G5D, chronic kidney disease treated by dialysis; VSMC, Vascular Smooth Muscle Cell; AGEs, advanced glycation end products; PBUT, Protein-bound uremic toxins.

In summary, CKD-G5D patients often have traditional risk factors for coronary heart disease, as well as some non-traditional risk factors (Fig. 1). These patients also have significantly increased levels of inflammatory factors, oxidation, and urinary toxins. This environment promotes the phenotypic transformation of VSMC, resulting in calcification and coronary atherosclerosis. Compared with the non-CKD population, the plaques formed in CKD-G5D patients are more unstable and more likely to result in an ACS.

## 4. Screening for CAD in CKD-G5D Patients

CAD accounts for a large proportion of the cardiovascular disease in CKD-G5D patients, with a several-fold higher incidence than in non-CKD patients. CKD-G5D patients also have worse outcomes after cardiovascular events. The hospitalization and long-term mortality rates of dialysis patients recorded in the Global Registry of Acute Coronary Events are three-fold higher than those of non-CKD patients [33]. Therefore, it is very important to screen CKD-G5D patients for CAD. However, the Framingham score prediction tool is suitable only for traditional risk factors. Because non-traditional risk factors also play an important role in CKD-G5D patients, the Framingham model has been estimated to underestimate the risk of CAD in CKD patients by 50% [34].

Although the prevalence of CAD is higher in patients with CKD-G5D, the typical symptoms are often absent, thus making it difficult to diagnose this condition from the clinical presentation only. There are likely to be several reasons for the apparent absence of symptoms. CKD-G5D patients are often seriously ill and their ability to exercise is very low, meaning they are unable to reach the exertion threshold for symptoms to appear. In patients with diabetes, the development of severe neuropathy in later stages may also mask the symptoms of angina pectoris. Compared with non-dialysis patients, the proportion of ST segment changes in patients with chest pain is also lower. CCS in CKD-G5D patients can manifest as exercise-induced chest discomfort, hypotension or arrhythmias [9, 35, 36]. In summary, screening for CAD in patients with CKD-G5D or even ESRD by clinical presentation is inaccurate because many patients present as either asymptomatic or with atypical symptoms.

Screening with serum biomarkers may be a viable alternative for CAD screening since it is non-invasive and cost-effective. Cardiac troponin (cTn) is a commonly used biomarker for the presence of myocardial necrosis [1, 37] and is the main predictor of increased all-cause mortality and cardiac death observed in dialysis patients. The sensitivity of cTn for predicting CAD in CKD-G5D patients is high. However, the specificity is much lower than in non-CKD patients because more than one third of CKD-G5D patients have long-term elevated troponin levels [38, 39, 40]. This necessitates the adjustment of critical values to improve specificity, while still ensuring high sensitivity. At present, there are no guidelines for the interpretation of cTn values in CKD-G5D patients in clinical practice. More research data is needed to help determine a suitable threshold. Other biomarkers also have the same problem of high false-positive rates, making them difficult to use in clinical practice. SuPAR may be a suitable biomarker, as its level is increased by common risk factors for CKD and CVD such as smoking, hypertension, and diabetes. The SuPAR level is associated with coronary and peripheral atherosclerotic disease. Moreover, it can predict renal and CVD outcomes across age, gender, ethnicity and clinical setting, independently of these risk factors [32].

Exercise testing is also not a good diagnostic indicator, since many CKD-G5D patients have abnormal electrocardiograms (ECGs) in the resting state [41]. The numerous co-morbidities found in advanced CKD patients such as left ventricular hypertrophy (LVH), electrolyte disorders, volume overload and anemia can give rise to abnormal ECGs, making these difficult to interpret. This limitation reduces the sensitivity and specificity of exercise testing from 68% and 77% respectively in the general population, to just 35% and 64% in CKD-G5D patients [41, 42]. In addition, many patients with late stage CKD have comorbidities of the motor and nervous systems. This makes it difficult to reach a defined activity threshold and requires drug stimulation [41] using dobutamine stress echocardiography (DSE). DSE and invasive coronary angiography (ICA) are used to detect and quantify coronary disease. Although ICA is better than DSE at predicting all-cause mortality [43], DSE is more effective at identifying high-risk groups. Although the accuracy of DSE is not particularly high, it is widely used since it is non-invasive and relatively safe [44, 45]. In summary, exercise testing is not an accurate method of screening for CAD in CKD-G5D patients. DSE may be a more accurate technique and has few contraindications.

The coronary artery calcium score (CACS) has the advantages of being fast, non-invasive, and having low radiation exposure. While CACS has high accuracy in the general population [46], up to 83% of dialysis patients have an increased score [47]. Therefore, the utility of this test for the CKD-G5D population is low. However, the negative predictive value of CACS is very good [48] and can help to exclude CAD in CKD-G5D patients.

Coronary computed tomography (CCTA) is also a good imaging tool. Its negative predictive value is very high and it has moderate positive predictive value [49]. CTCA relates to the patients’ calcium load, and hence to the specificity of diagnosis [50, 51, 52]. Therefore, it should be used together with CACS to select patients with a zero to low calcium load. However, because the incidence of CACS in CKD-G5D patients is significantly increased, the utility of CCTA in these patients is limited.

Myocardial perfusion single-photon emission computed tomography (MPS) is a non-invasive nuclear imaging technique [53] that may give predictive information on the CKD-G5D population. Studies have shown that abnormal MPS results are independent predictors of mortality [54, 55]. MPS is primarily used to predict mid-to-high probability coronary events in the general population, and has only moderate predictive accuracy in the CKD-G5D population. Nine studies with a total of 582 CKD-G5D subjects were included in a Cochrane meta-analysis, with the overall sensitivity and specificity for angiographic CAD found to be 0.74 and 0.70, respectively [56].

ICA is the gold standard technique for detecting coronary artery stenosis, but has the disadvantages of being expensive, invasive and associated with some risks [57]. In addition, ICA can overestimate the clinical significance of CAD [58], since anatomical stenosis may not equate to functional stenosis [59]. There is also a slightly increased risk for CKD-G5D patients due to the invasive nature of the ICA procedure. More clinical data are needed to comprehensively evaluate the benefits and risks of ICA in the CKD-G5D population.

In summary, several methods are available for CAD screening in CKD-G5D patients. ICA works well, but its cost and associated risks need to be considered. Among the non-invasive methods, exercise testing is not widely used, and DSE needs to be properly applied. The positive detection rates of MPS and serum cTn are satisfactory, but these methods are limited by a high false-positive rate. CACS and CCTA both have high negative prediction value, but the positive prediction accuracy of CACS is low, and CCTA is greatly affected by the calcium load. At present, an ideal testing method is still lacking, with each of the current methods having advantages and disadvantages. There is a need to combine various screening methods in order to match the specific clinical condition of each patient and to reduce the rates of false positives and false negatives. The advantages and disadvantages of each diagnostic method discussed above are summarized in Table 1.

**Table 1.** **Advantages and disadvantages of CAD screening methods in CKD-G5D patients**.

Method	Advantages	Disadvantages
Serum biomarkers	rapid and inexpensive	high false-positive rates
Exercise test	non-invasive	abnormal ECG in the resting state; low motor ability
DSE	non-invasive and harmless	low accuracy
ICA	good accuracy	invasive; expensive
CCTA	high negative predictive value	affected by patients’ calcium load
CACS	fast, non-invasive and low radiation amount	normally high in CKD-G5D
MPS	non-invasive	low predictive power in the CKD-G5D population

Abbreviations: CAD, coronary artery disease; ECG, electrocardiogram; CKD-G5D, chronic kidney disease treated by dialysis; DSE, dobutamine stress echocardiography; ICA, invasive coronary angiography; CCTA, coronary computed tomography; CACS, coronary artery calcium score; MPS, myocardial perfusion single-photon emission computed tomography.

## 5. Management and Treatment Strategies for CAD in CKD-G5D Patients

The small number of clinical trials involving the CKD-G5D population means there is insufficient evidence to support various management and treatment strategies for CAD. The treatment standards and methods used in the general population are generally not suitable for the CKD-G5D population. The incidence of complications due to the lack of detection or treatment in the dialysis population tends to increase as the sensitivity and specificity of the various detection methods for CAD in this population decrease. The choice of diagnostic method for dialysis patients who suffer from CAD needs careful consideration, together with decisions on whether to undergo invasive studies or coronary artery bypass grafting (CABG), and the choice of appropriate drug treatment plan.

Although drug therapy is the basis of CAD treatment, the few clinical studies performed so far mean the type of drug therapy for patients with advanced CKD is unclear, with most of the guidelines having been derived from trials on early CKD [60]. The use of statins in these patients is controversial. Some studies suggest the benefits from statin-based treatment (reduction of major cardiovascular events) decrease with declining GFR, with little evidence to show that dialysis patients benefit from these drugs [9, 60]. However, other studies have reported that statins prevent the development of endothelial dysfunction caused by acute inflammation in hypercholesterolemic patients [61], as well as slowing the increase in aortic stiffness of CKD patients [62]. The guidelines from “Kidney Disease: Improving Global Outcomes” advise against the use of statins in dialysis patients [63]. In contrast, the heart and renal protection (SHARP) trial concluded that statins reduce the incidence of atherosclerosis with equal effect in dialysis and non-dialysis patients [64]. However, the number of dialysis patients in the SHARP trial was small, and additional clinical studies are required to confirm the effect of statins in CKD-G5D patients. A large retrospective cohort study found that dialysis patients with peripheral arterial disease who were treated with statins had a lower risk of amputation and of central vascular and all-cause death compared with untreated patients [65]. The authors concluded that statin therapy may have a protective effect on patients with renal failure and peripheral arterial disease who receive long-term maintenance dialysis. There is currently a lack of guidance from various professional societies regarding the use of statins in CKD-G5D patients [66, 67]. All antihypertensive agents passively reduce arterial stiffness through BP-dependent mechanisms, including reduction of arterial wall stretch [19]. However, some antihypertensive drugs can reduce arterial stiffness but have little effect on BP, including angiotensin-converting enzyme inhibitors, angiotensin receptor blockers, and direct renin inhibitors [68, 69]. β-blockers appear to be less effective than other types of antihypertensive drugs in terms of reducing central BP and arterial stiffness [70]. Anti-inflammatory drugs can reduce chronic inflammation, which in some patients is beneficial for reducing arterial stiffness. Long-term use of anti-tumor necrosis factor therapy has been reported to restore aortic arteriosclerosis to levels similar to a matched control group [71, 72]. Although anti-inflammatory drugs can be used to reduce uremic toxins and chronic inflammation in CKD patients, they are not recommended because of adverse effects on renal function. Moreover, there is a lack of data on the impact of anti-inflammatory drugs on arterial stiffness in CKD patients [19, 73]. Antioxidants such as ascorbic acid and asymmetric dimethylarginine can improve blood flow-mediated dilation in CKD patients and reduce central BP [74, 75]. There is also evidence that allopurinol, a drug that reduces the plasma level of uric acid, can protect endothelial function in patients with or without CKD. The administration of allopurinol significantly reduced the pressor index, but did not reduce arteriosclerosis [76, 77]. Arterial stiffness in patients with ESRD can be reduced by phosphate binders that do not contain calcium, such as Villam hydrochloride, which reduce phosphate absorption in the gastrointestinal tract [78]. However, similar results were not reported in patients with early stage CKD [79]. Indeed, it may take several years to reverse the structural arteriosclerosis caused by VC. In summary, the drug treatment scheme for CKD-G5D patients is relatively complicated and there are currently no specific guidelines for the use of any of the above medications in these patients. This is because the metabolic status of CKD-G5D patients and their complications are significantly different to those of early CKD patients. Moreover, the related clinical trial data on CKD-G5D patients is still scarce.

Drug therapy for CKD-G5D patients can result in several complications, including the development of thromboembolism. Anti-thrombotic therapy includes a combination of anticoagulant and antiplatelet drugs to reduce the risk of ischemia and thromboembolism, but this comes at the cost of increased bleeding events. Direct oral anticoagulant (DOAC) is the most commonly used anticoagulant therapy, but there is controversy over whether to use DOAC or vitamin K antagonist (VKA) [80]. As the GFR decreases with age, the risk of bleeding is increased. DOAC undergoes varying levels of renal elimination (approximately 80% of dabigatran, 36% of rivaroxaban, 27% of apixaban, and 50% of edoxaban). It can therefore accumulate in patients with decreased renal function, and dose adjustment is recommended. Although 50–60% of dabigatran can be removed in a single dialysis, other DOAC components are more difficult to remove because of their strong binding to plasma proteins [80, 81]. The safety of DOAC in CKD patients is uncertain, especially in ESRD patients with severely impaired renal function. On the other hand, an increased incidence of atrial fibrillation (AF) has been reported in ESRD patients with CKD [82, 83, 84], and DOAC is associated with an increased risk of bleeding events in these patients. AF in patients with CKD has been associated with worsening renal function and progression to ESRD. Lower GFR has also been associated with higher risks of major and non-major bleeding events in patients taking oral anticoagulants. For these reasons, careful monitoring of renal function is recommended in such patients. The restricted use of DOAC in CKD-G5D or ESRD patients needs to be carefully reviewed, especially because of the lack of compelling evidence to guide clinical decisions [85]. Currently, DOAC is generally not recommended for patients with CKD-G5D or ESRD, with warfarin (the most commonly used VKA) being favored instead. A recent review has summarized the relevant clinical trials for current antithrombotic treatment strategies [83]. Triple antiplatelet therapy (TAPT) exposes AF patients to a high risk of bleeding during 30-day follow-up. Several randomized clinical trials involving about 12,000 patients showed that dual antiplatelet therapy (DAPT) can significantly reduce bleeding events compared with TAPT. Observational studies have also shown that TAPT is still mainly a prescription, whereas DAPT is used only in patients considered to be at high risk for bleeding. Therefore, TAPT is not suitable for dialysis patients with known CAD. With regard to antiplatelet agents, dialysis patients are at significant risk for both bleeding and thrombosis. This creates a major dilemma when choosing the best antiplatelet therapy to manage ACS in this high-risk population. Current ESC guidelines recommend DAPT (combined with aspirin and a potent ${\mathrm{P2Y}}_{12}$ inhibitor) for patients with ACS undergoing percutaneous coronary intervention (PCI) [86]. However, there is no consensus on the specific dosing due to the limited number of clinical trials in the HD population. Recent studies have shown that early discontinuation of aspirin in HD patients may reduce bleeding complications without increasing the risk of ischemic events. Although there was no significant change in all-cause mortality with early discontinuation of aspirin, the risk of major bleeding was significantly reduced. The ${\mathrm{P2Y}}_{12}$ inhibitor clopidogrel is more widely used, but studies in dialysis patients have shown that ticagrel may inhibit platelets faster and more strongly than clopidogrel [87].

Patients with ESRD on maintenance HD have many comorbidities, including unstable angina pectoris. Blocking the inward sodium channel with ranolazine has been shown to reduce the incidence of stable angina in patients with chronic CAD, but its use in dialysis patients is still debatable. A ranolazine plasma protein binding rate of 62% may not be eliminated by dialysis, and therefore a reduced dose of ranolazine is generally recommended for dialysis patients [88, 89].

Among the relatively new therapeutic drugs for CKD, sodium-dependent glucose transporter 2 inhibitor (SGLT-2i) and finerenone appear to give improved cardiovascular outcomes. It is important to reduce blood glucose and cardiovascular events in patients with advanced CKD accompanied by diabetes. Recent guidelines from the American Diabetes Association recommend medications with cardiovascular benefits for patients with Type-2 diabetes mellitus (T2DM) and atherosclerotic CVD. In a series of clinical trials on the cardiovascular safety of hypoglycemic drugs, SGLT-2i was found to reduce major adverse cardiovascular events and to have protective effects for non-diabetic patients with HF. The latest DAPA-CKD clinical trial demonstrated benefit from dapagliflozin in CKD patients, both with or without T2DM [90]. In addition, the EMPEROR-Reduced and DAPA-HF trials demonstrated that SGLT-2i reduced both the risk of hospitalization for heart failure and cardiovascular death in T2DM patients [91]. In addition, SGLT-2i slowed the decline in renal function in patients with or without heart failure, and decreased the ejection fraction. SGLT-2i clearly has protective properties for cardiac and renal function in patients with CKD. The FIDELIO-DKD trial randomized 5734 patients with T2DM and CKD in a 1:1 ratio to receive either fenidone (2833 subjects) or placebo (2841 subjects) [92]. The primary endpoint (>40% decrease in GFR, or death due to renal disease) incidence was 17.8% in the fenidone group and 21.1% in the placebo group (hazard ratio [HR]: 0.82; 95% confidence interval [CI]: 0.73–0.93; *p* = 0.001). The combined outcome rate for cardiovascular events was 13.0% in the fenidone group and 14.8% in the placebo group (HR: 0.86; 95% CI: 0.75–0.99; *p* = 0.03). Hence, these studies show that fenidone is effective in delaying the progression of CKD and in reducing cardiovascular events in patients with end-stage diabetic nephropathy and CKD. Although such large-scale studies have provided guidance for the use of these two new drugs, more research is needed to determine their benefits in CKD-G5D patients.

For CKD-G5D patients with ACS, it remains controversial whether complete revascularization with PCI or CABG, or standard medical treatment is the best course of action. Studies of asymptomatic patients without CKD or ESRD have failed to show that revascularization is beneficial for outcome. Long-term mortality after preventive coronary revascularization in clinically stable coronary heart disease patients is similar to those receiving the best drug treatment (23% vs. 22%, *p* = 0.92) [60]. Hence there is no clear evidence that early invasive treatment is beneficial in this population. The current status of management and treatment schemes for several specific CAD classifications are reviewed in the following section.

CCS is usually atypical in dialysis patients and may be difficult to differentiate from dialysis symptoms. It therefore requires particular attention in CKD-G5D patients. The ESC published diagnosis and management guidelines for CCS in 2019 [7]. These recommend regular electrocardiograms and more advanced, non-invasive tests in patients with a high cardiovascular risk as defined by the risk distribution map of ESC-SCORE. For CKD-G5D patients, special attention should be paid during risk assessment because late-stage CKD is itself one of the risk factors. This means that other relatively controllable risk factors such as smoking should be reduced or eliminated as much as possible. However, the efficacy of non-invasive stress testing in patients with CKD-G5D is also unsatisfactory, as previously discussed. The International Study of Comparative Health Effectiveness with Medical and Invasive Approaches (ISCHEMIA) study randomized patients into two groups: one received revascularization plus optimal drug treatment, and the other received only the optimal drug treatment. No significant difference in treatment effect was observed between the two groups. ISCHEMIA-CKD is a substudy of the patients with advanced CKD. This researcher-initiated, international randomized trial aims to determine whether coronary angiography and revascularization (PCI or coronary artery bypass grafting (CABG)) combined with the drug therapy recommended by the guidelines can reduce cardiovascular events in patients with advanced CKD and moderate or severe myocardial ischemia. Results from the 2019 study showed that the probability of death or myocardial infarction at 2.3 years follow-up was 36.4% in the combined treatment (invasive) group and 36.7% in the drug-treated (conservative) group (*p* = 0.95) [93]. These results were confirmed in the latest follow-up study published in 2022, which found a similar rate of progression to dialysis treatment between the two groups [94], although the median time to dialysis was considerably shorter in the invasive treatment group. The above trial findings suggest that early revascularization in asymptomatic CKD-G5D patients with stable CCS may not confer additional therapeutic benefit compared to drug therapy. Currently, there are insufficient clinical trials to be definitive about the best medical therapy for CKD-G5D patients with CCS. Anti-hypertensive drugs have generally been used as first-line therapy. Beta-blockers are widely used and can significantly improve the outcome of high-risk groups [95, 96]. Angiotensin receptor blocker (ARB) or angiotension converting enzyme inhibitors (ACE-I) are recommended for dialysis patients with hypertension [97]. More clinical research and guidance from specialized societies are needed to determine the role of statins in these patients.

The diagnosis of non-ST-segment elevation myocardial infarction in CKD-G5D patients is also difficult because they have atypical clinical symptoms and may present with nonspecific ECG changes. The baseline level of cardiac troponin T (cTnT) gradually increases with the development of CKD. A higher cTnT threshold should therefore be used for the diagnosis of acute myocardial infarction (AMI) in dialysis patients. Studies have suggested that CKD-G5D patients with non-STEMI can benefit from PCI, and have a better outcome than drug therapy alone [98, 99]. However, there are currently no clear recommendations on the timing of interventions for these patients, or the strategy of revascularization. ESC guidelines from 2015 suggest that dialysis patients should undergo invasive methods in order to make decisions regarding early intervention. However, several meta-analyses that support this view did not include CKD-G5D patients. A systematic review by Shaw *et al*. [100] in 2016 did not support early invasive treatment for patients with dialysis or renal transplantation.

Evidence-based guidance is also urgently needed for the treatment of CKD-G5D in patients with STEMI. This complex patient group is not well represented in STEMI trials, and there are few studies on this cohort. Cardiologists have so far failed to reach a consensus on management and treatment plans for this population. The diagnosis of STEMI in CKD-G5D patients is difficult, the presentation is atypical, and there are more complications resulting in higher mortality and poor prognosis. Moreover, the risk of invasive coronary revascularization and drug treatment is higher than in the general population. Dialysis patients usually receive less reperfusion therapy, thrombolysis therapy, statins, PCI and CABG compared with the general population [101]. In a 2017 cohort study of 30,072 CKD-G5D patients with STEMI, 65.2% received reperfusion therapy, 2.1% thrombolysis therapy, 50.5% coronary angiography, 32.2% PCI, and 6.3% CABG [101]. The most recent ESC STEMI guidelines issued in 2017 recommend that renal function in STEMI patients should be independently and rapidly evaluated, regardless of whether or not the patient receives reperfusion treatment [102]. Due to the lack of clinical trial data, this guideline did not provide complete and specific indications for reperfusion treatment in CKD-G5D patients. However, the ESC recommends reperfusion therapy for patients with STEMI diagnosed within 12 hours [103]. If PCI cannot be performed in time after the diagnosis is established, thrombolysis should be performed within 12 hours, since the probability of massive hemorrhage in the CKD-G5D population is no higher than in the general population. Observational studies have shown that PCI is more effective than thrombolysis [104]. PCI is associated with better short-term survival than CABG, but worse long-term survival. Drug therapy guidance for this population is limited, but currently aspirin and heparin appear to be preferred. DAPT is used to reduce the incidence of thrombosis and AMI following hospital discharge after invasive treatments such as PCI and coronary stenting. However, DAPT can also increase the risk of bleeding events [102]. The ESC guidelines use a score for hemorrhagic risk with DAPT following PCI. This comprises the 5 indicators of age, GFR, hemoglobin, white blood cell count, and previous bleeding events. Short-term DAPT (3–6 months) is recommended when the score is >25, while long-term DAPT (12–24 months) is recommended when the score is <25. Patients with CKD-G5D have little to no renal function, and hence their score should start at 25 [105]. A cohort study concluded that the risk ratio was lower in the long-term DAPT group. However, this study included GFR <40 mL/min, and the number of CKD-G5D patients was unknown. Therefore, the conclusion reached may not apply to CKD-G5D patients [105]. A similar study from Taiwan compared the outcomes of DAPT treatment for duration of longer or shorter than 6 months and concluded that length of treatment was not related to patient outcomes [106]. In conclusion, there is still a dearth of reliable data on treatment outcomes from DAPT regimens in CKD-G5D patients with STEMI following invasive treatment. Since the conclusions of many studies are contradictory, it is still too early to establish guidelines on this topic.

In view of the complexity of current treatments, we created a simple table to list the different treatment categories, the treatment objective, and the current evidence concerning outcomes (Table 2).

**Table 2.** **Main effects of common treatments**.

	Treatment	Major objective	Remarks
Medication	Statin	There is no evidence of benefit	Disputable benefit
	Anti-hypertensive drug	Reduce arterial stiffness	
	Anti-inflammatory agent	Reduce chronic inflammation	Not recommended: impaired renal function; lack of data on vascular effects
	Antioxidant drugs	Improve arterial condition	Ascorbic acid: improve dilation
	Phosphate binder	Reduce gastrointestinal phosphate absorption while improving arterial stiffness	The effect is not obvious due to a lack of data
	Allopurinol	Protect endothelium	
	DOAC	Treatment of thromboembolic complications	Difficult to clear by dialysis, so not suitable for renal insufficiency or dialysis patients
	Ranolazine	Treatment of angina pectoris complications	Reduction is required
	SGLT-2i	Treat diabetes	Cardiorenal protection
	Finerenone	Cardiovascular protection	
	VKA	Commonly used in DAPT or TAPT, no less effective than DOAC for HD patients	Mainly Warfarin
	SAPT	Insufficient effect on HD patients	
	DAPT	More applicable to HD patients	Lack of guidance on specific medication regimens for this specific population of HD patients
	TAPT	Relatively high risk of hemorrhage, not well suited for HD patients	Assessment of patients at low risk of bleeding allows for individualized application
Invasive therapy	PCI	Treat angina pectoris and myocardial ischemia	At present, the research shows that early invasive treatment does not result in obvious improvement, and the outcome is not as good as with drug treatment
	CABG	Blood supply reconstruction	Same as above

Abbreviations: HD, hemodialysis; DOAC, direct oral anticoagulant; SGLT-2i, sodium-dependent glucose transporter 2 inhibitor; VKA, vitamin K antagonist; SAPT, single-antiplatelet therapy; DAPT, dual-antiplatelet therapy; TAPT, triple-antiplatelet therapy; PCI, percutaneous coronary intervention; CABG, coronary artery bypass grafting.

## 6. Conclusions

The incidence of CVD in the CKD-G5D population is high and is the leading cause of death in these patients. In 2016 in the United States, the mortality rate for all dialysis patients was 179/1000 patient-years, 37% of which was attributed to cardiac causes [107, 108]. Sudden death is common in CKD-G5D patients, probably due to changes in volume, electrolytes, and drug concentrations that trigger arrhythmias in those with myocardial disease (LVH and heart failure) [9, 109]. As the GFR decreases, non-arterial events account for a higher proportion of CVD events.

The incidence of CAD increases linearly with the progression of CKD, as CKD is itself a risk factor for CAD. The CKD population has a high incidence of traditional risk factors for CAD (e.g., diabetes, hypertension), as well as non-traditional factors such as oxidative stress, chronic inflammation, and VC. Because of these co-morbidities, CAD progresses faster in CKD-G5D patients than in the general population. Screening for coronary heart disease in these patients is also challenging. As CKD progresses, the clinical manifestations of CAD patients are often atypical. For example, only 44% of dialysis patients compared to 68% of non-dialysis patients suffer from chest pain when an AMI occurs [9]. In addition, the risks associated with invasive examinations are also increased in CKD-G5D patients. Therefore, it is difficult to reach a consensus on the management and treatment strategies for this complex patient population. The use of drug treatment for coronary heart disease is usually less in CKD patients than in non-CKD patients. However, the percentage of patients receiving treatment has increased in recent years. The benefits of invasive treatment in CKD patients are still controversial, with some authors reporting that early intervention is beneficial, whereas others have claimed there are no benefits compared to conservative drug treatment. It has proven difficult to reach a consensus in this field due to the lack of relevant clinical trial data and the limited number of CKD-G5D patients enrolled in many of the studies. More data from large-scale clinical studies are needed to confidently guide the diagnosis and treatment of this complex group of CAD patients.
